# Checklist of Ornamental Trees, Shrubs, and Succulents of Apulia (Southern Italy)

**DOI:** 10.3390/plants13172463

**Published:** 2024-09-03

**Authors:** Giuseppe Venturella, Emilio Di Gristina, Raimondo Pardi, Fortunato Cirlincione, Maria Letizia Gargano

**Affiliations:** 1 Department of Agricultural, Food and Forest Sciences, University of Palermo, Viale delle Scienze, Bldg. 5, 90128 Palermo, Italy; giuseppe.venturella@unipa.it; 2 NBFC, National Biodiversity Future Center, 90133 Palermo, Italy; 3 Department of Soil, Plant and Food Sciences, University of Bari Aldo Moro, Via G. Amendola, 165/A, 70126 Bari, Italy; fortunato.cirlincione@uniba.it (F.C.); marialetizia.gargano@uniba.it (M.L.G.)

**Keywords:** urban street trees, historic gardens, parks, private gardens, Apulia, southern Italy

## Abstract

In this study we focused on the need to fill a knowledge gap among Italian botanical studies namely that of ornamental species census. In particular, we addressed one of the regions in southern Italy with less knowledge in the field of such studies and with an obvious presence of non-native species. A widespread census of the Apulian territory was carried out between 2021 and 2024 in both urban and suburban areas including street trees, parks, and private and historic gardens. The inventory of ornamental trees, shrubs, and succulents of Apulia (southern Italy) was carried out in six provinces, i.e., Bari, Barletta-Andria-Trani, Brindisi, Foggia, Lecce, and Taranto. The checklist comprises 287 taxa (including 265 species, 6 varieties, 5 subspecies, and 11 forms) included in 179 genera belonging to 78 families. We evaluated the number of taxa per families and genera, the presence of each taxa in the provinces of Apulia, the number of taxa per occurrence status, growth forms, geographical origin, and the number of native and alien taxa and also the artificial hybrids. Remarks of the most significant taxa and evaluation of geographical distribution in Italy were also taken into consideration. A large number of surveyed taxa (51.74%) are comprised in the list reported in the recent study on allochthonous vascular flora in Italy with a marked prevalence of Neophyte Casual Alien and Neophyte Naturalized Alien species.

## 1. Introduction

Ornamental plants are important elements of urban areas useful in improving air quality, water quality, climate regulation, nutrient recycling, and pollination. Within urban parks and historic gardens, they also play a cultural role by providing recreational opportunities for citizens resulting in improved well-being [1].

Ornamental plants are also used in the form of city street trees for aesthetic beauty but also to help reduce air pollution and temperature by shading asphalt and buildings with a direct impact on Urban Heat Islands [2]. Some of these ornamental species are of particular aesthetic value and, at times, of considerable age and therefore considered by citizens as part of the urban landscape.

On the other hand, the prevalence of alien ornamental species over native species leads to a number of critical issues that have been studied by several authors [3,4,5]. Many of the invasive plants recorded in Italy were intentionally introduced for ornamental reasons than for any other purpose.

The number of allochthonous species is gradually and steadily increasing, and the phenomenon of biological invasions is known to cause damage to biodiversity, ecosystem services, human health, and the economy [6,7]. This is in line with the growing trend of biological invasions resulting from the intentional or accidental introduction of species as ornamentals by humans into areas outside their native range. Anthropogenic factors play a key role in the establishment and subsequent spread of alien species. Emblematic is the example of *Ailanthus altissima* (Mill.) Swingle, a tree that belongs to the large group of ornamental trees dispersed by wind or birds, which is an important management problem for the conservation of cultural heritage sites [4]. Also not to be underestimated is the spread of novel plant diseases through the introduction of alien ornamental species both in nurseries and within urban centers [8,9].

However, the analysis of urban street trees is often overlooked, although they are key green elements within cities, because of their specific ecological and socio-economic functions [10]. In Italy, previous studies have dealt marginally with ornamental species, and some references can be found in papers dealing more generally with allochthonous vascular flora or alien species [11]. On the contrary, reports from other countries demonstrated a wide interest in ornamental plants as a source of multiple ecosystem services, including their utility in the phytoremediation of contaminated soil, air, and water [12], their capacity to remove volatile organic compounds from indoor air [13], and their role as sinks and bioindicators [14], as well as for the analysis of the toxicity of many ornamental shrubs used for their aesthetics due to accidental ingestion [15], etc.

In 2021, we launched a census of ornamental plants in Apulia, a region in southern Italy, with the aim of providing an inventory of trees, shrubs, and succulents grown in street trees, parks, private gardens, and historic villas.

The unpublished data collected during the census are the subject of this survey which provides an important contribution to the knowledge of an important aspect of the alien and native flora of Apulia.

A further purpose of this research is to highlight the presence of ornamental species in the Apulian territory, an under-researched area of botanical studies in the region. The objective of this study is to provide a list as close to reality as possible of the ornamental species in Apulia that will allow subsequent assessments by local governments of the aesthetic value of the plants but also of the possible risks related to invasiveness and public health mainly in terms of increased allergies.

## 2. Results

A total of 287 taxa (including 265 species, 6 varieties, 5 subspecies, and 11 forms) included in 179 genera belonging to 78 families were surveyed in the six provinces of the Apulia region, i.e., Bari, Barletta-Andria-Trani, Brindisi, Foggia, Lecce, and Taranto.

**Figure 1:**
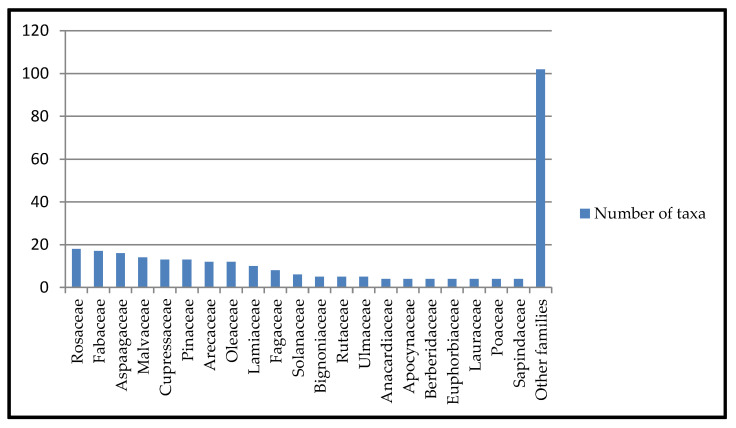
Number of taxa per family based on taxa surveyed in the Apulian territory.

The highest number of taxa (Figure 1) is found in the families Rosaceae (18 taxa), Fabaceae (17 taxa), Asparagaceae (16 taxa), Malvaceae (14 taxa), Cupressaceae (13 taxa), Pinaceae (13 taxa), Arecaceae (12 taxa), Oleaceae (12 taxa), Lamiaceae (10 taxa), Fagaceae (8 taxa), Solanaceae (6 taxa), Bignoniaceae (5 taxa), Rutaceae (5 taxa), Ulmaceae (5 taxa), Anacardaceae (4 taxa), Apocynaceae (4 taxa), Berberidaceae (4 taxa), Euphorbiaceae (4 taxa), Lauraceae (4 taxa), Poaceae (4 taxa), and Sapindaceae (4 taxa).

The largest number of taxa is found in the genera *Quercus* L. (eight taxa), *Prunus* L. (seven taxa), *Tamarix* L. (seven taxa), *Pinus* L. (six taxa), *Asparagus* Tourn. ex L. (five taxa), *Ficus* Tourn. ex L (five taxa), *Ligustrum* L. (five taxa), *Ulmus* L. (five taxa), *Brachychiton* Schott and Endl. (four taxa), *Citrus* L. (four taxa), *Euphorbia* L. (four taxa), *Salvia* L. (four taxa), and *Yucca* L. (four taxa).

A total of 31.2% (90 taxa) of the taxa listed in Appendix A Table A1 are found in all six provinces where the census was conducted, while 103 taxa (35.76%) have only been surveyed in one province.

**Figure 2:**
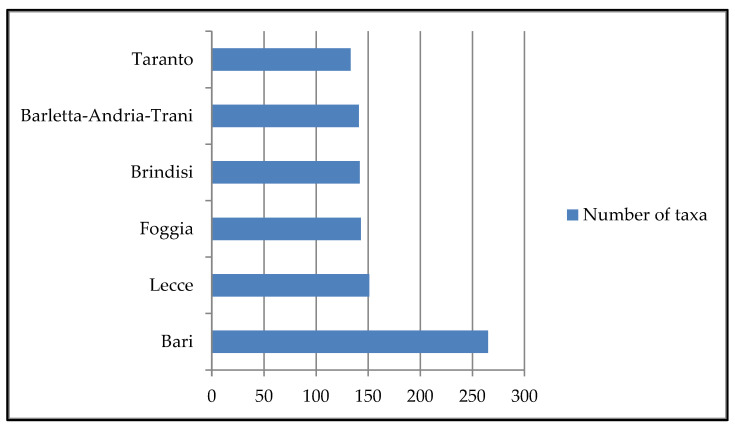
Number of taxa surveyed in each Apulian province.

Bari (264 taxa) is the province in Apulia with the largest number of taxa surveyed (Figure 2) followed by Lecce (151 taxa), Foggia (143 taxa), Brindisi (142 taxa), Barletta-Andria-Trani (141 taxa), and Taranto (133 taxa).

**Figure 3:**
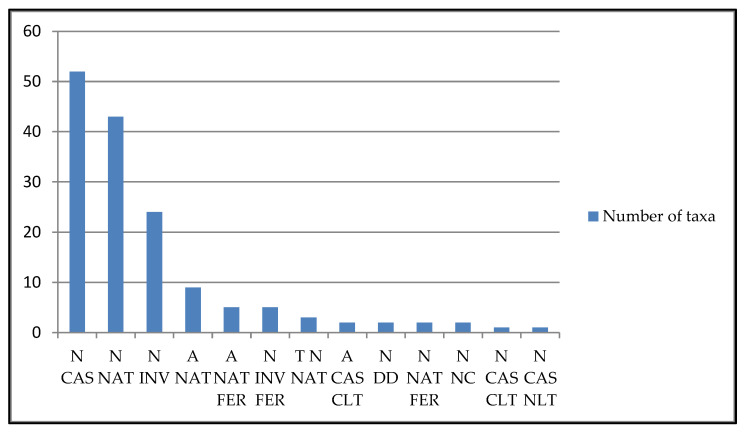
Number of taxa per occurrence status (sensu Galasso et al. [17]) based on taxa surveyed in the Apulian territory.

A large number of the surveyed taxa (51.74%) are included in the list reported in the recent study on allochthonous vascular flora in Italy [16]. According to Galasso et al. [17], most of them fall into the category Neophyte Casual Alien (N CAS) (52 taxa) followed by Neophyte Naturalized Alien (N NAT) (43 taxa), Neophyte Invasive Alien (N INV) (24 Taxa), Archeophyte Naturalized Alien (A NAT) (9 taxa), Neophyte Invasive Alien Feral (N INV FER) (5 taxa), Archeophyte Invasive Alien Feral (A NAT FER) (5 taxa), Taxonomically Doubtful Neophyte Naturalized Alien (T N NAT) (3 taxa), Neophyte Naturalized Alien Feral (N NAT FER) (2 taxa), Archeophyte Casual Alien Culton (A CAS CLT) (2 taxa), Neophyte Unclear Alien Status (N NC) (2 taxa), Naturalized Data Deficient N DD (2 taxa), Neophyte Casual Alien Culton (N CAS CLT) (1 taxa), and Neophyte Casual Alien Naturalized (N CAS NLT) (1 taxa) (Figure 3).

**Figure 4:**
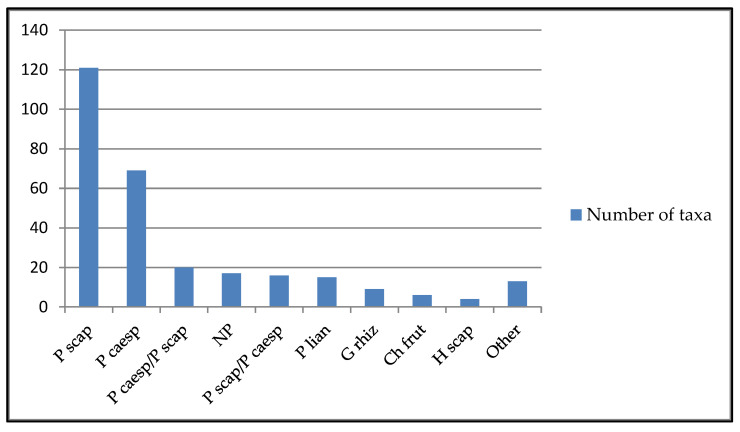
Number of taxa per biological form categorized according to *Plants of the World Online* (POWO 2024).

Based on what can be gleaned from “*Plants of the World Online*” edited by the Royal Botanic Gardens, Kew, among the plants surveyed, scapose phanerophytes (P scap) clearly predominate followed by caespitose phanerophytes (P caesp) (Figure 4) while there is a clear prevalence of Asian, African, and Central and South American geographic origins over European ones (Appendix A Table A2). From Appendix A Table A2, it is possible also to see the clear predominance of alien species (217 taxa) over native species (60 taxa) and 10 artificial hybrids (AH).

Most of the species surveyed are also commonly used as ornamentals in the other regions of Italy.

Taxa at the upper limit of their distribution in Italy are *Bauhinia variegata* L. var. *candida* Voigt, *Brachychiton* sp. pl., *Brahea armata* S. Watson, *Ceiba speciosa* (A. St.-Hil., A. Juss., and Cambess.) Ravenna, *Erythrina crista-galli* L., *Erythrina herbacea* L., *Euphorbia ingens* E. May. ex Boiss, *Euphorbia murielii* N.E.Br, *Euphorbia tirucalli* L., *Ficus* sp. pl., *Hibiscus* ×*rosa-sinensis* L., *Jacaranda mimosifolia* D. Don, *Musa ×paradisiaca* L., *Persea indica* (L.) Spreng, *Sabal palmetto* (Walter) Lodd. ex Schult. and Schult.f., *Sideroxylon spinosum* L., and *Yucca rostrata* Engelm. ex Trel.

*Erythrina crista-galli* and *Jacaranda mimosifolia* are species of high ornamental value that are fairly common but have only been expanding in southern Italy for a few years and, moreover, only locally.

Widespread in the Apulian provinces is the use of plants providing both productivity and ornamental value such as *Annona cherimola* Mill., *Citrus ×limon* (L.) Osbeck, *Citrus reticulata* Blanco, *Citrus ×sinensis* (L.) Osbeck, *Corylus avellana* L., *Feijoa sellowiana* (O. Berg) O. Berg, *Ficus carica* L., *Juglans regia* L., *Musa ×paradisiaca* L., *Punica granatum* L., *Prunus domestica* L., and *Prunus dulcis* (Mill.) D.A. Webb.

Moreover, *Sideroxylon spinosum* is of particular botanical interest providing ecological services and socioeconomic value. Its fruits provide an edible and marketable oil for cooking, cosmetic, and medicinal purposes. The vigorous tree cultivated in the University Campus of Bari is one of the few plants to date grown in Italy [18].

Another plant of application interest is *Euphorbia tirucalli* whose oil obtained from the modified stems and leaves is a valuable alternative energy source for biofuel production in some Arab and African countries [19].

**Figure 5:**
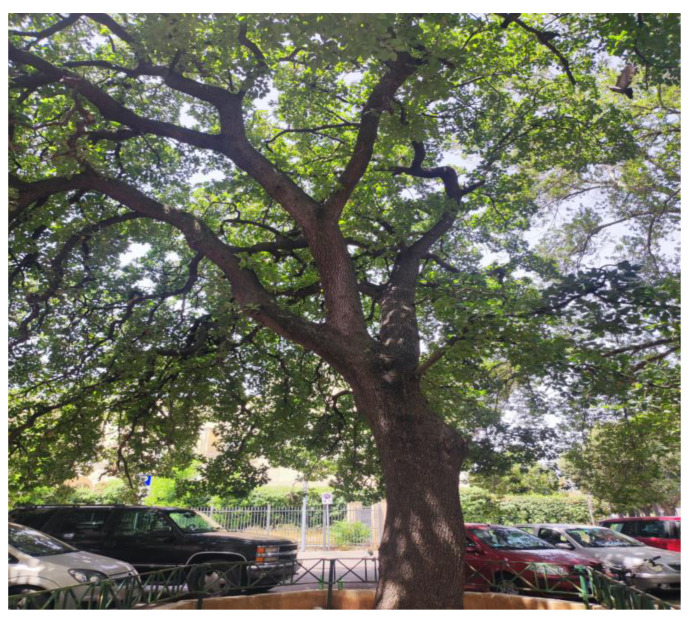
Monumental tree of *Quercus ithaburensis* subsp. *macrolepis* in the city of Lecce.

Finally, extensive use of species of the genus *Quercus* L. is noted. *Quercus ilex* L. is widely used in the streets of the city of Lecce where the crowns of the trees make contact with each other forming tunnels that provide coolness on hot days for citizens. In the city of Lecce, it is also possible to observe a monumental tree of *Quercus ithaburensis* subsp. *macrolepis* (Kotschy) Hedge and Yalt, (Figure 5), a species found in Italy only in Apulia and more precisely in Salento, mainly in the province of Lecce, and to a lesser extent in the province of Bari and Brindisi. This is a relict species from an old botanical garden that was dismantled in the early 20th century. Since 1993, *Q*. *ithaburensis* subsp. *macrolepis* is included in the Red List of the endangered botanical species in Italy. At the international level, the need to establish reserves to safeguard its genetic heritage has been recognized.

Trees of *Quercus pubescens* Willd. are used as ornamentals in the cities of Bari and Foggia.

Instead, *Quercus cerris* L., *Q*. *petraea* (Matt.) Liebl, *Q*. *trojana* Webb, *Q*. *robur* L., *Q*. *suber* L.*,* and *Q*. *trojana* Webb are used in the newly planted gardens in the city of Bari.

**Figure 6:**
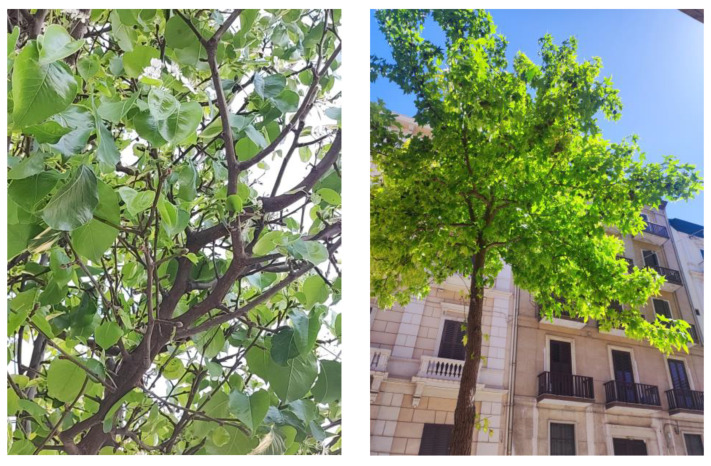
*Pyrus calleryana* (**left**) and *Liquidambar styraciflua* (**right**), two species widely used as ornamentals along the streets of Bari and Taranto.

In the cities of Bari and Taranto, among the newly planted species (Figure 6), we found *Liquidambar styraciflua* L., a medium-to-large tree, which is an excellent choice as it can provide protection from the sun’s rays in summer. Also, from a decorative point of view, it provides distinctive color effects in autumn thanks to the golden-yellow, orange, or red color of its leaves. In addition, it is a fast-growing species that does not require special care. On the contrary, the choice to plant *Pyrus calleryana* Decne is open to criticism since in Canada it is considered an invasive species, demonstrating the not-always prudent choices made by municipal administrations.

**Figure 7:**
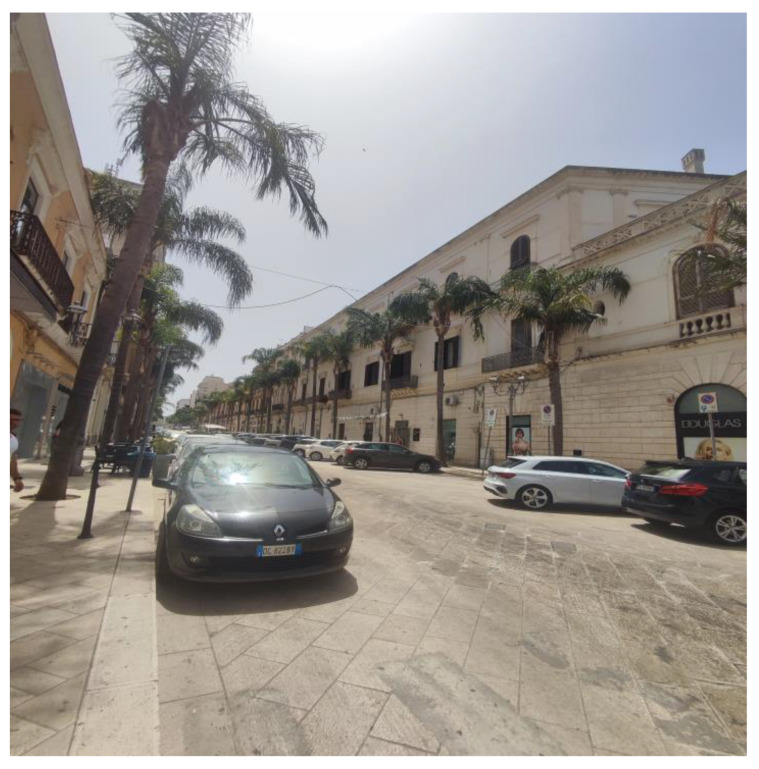
An unusual row of *Syagrus romanzoffiana* along the streets of Barletta.

Finally, among the various palms of ornamental use, *Syagrus romanzoffiana* (Cham.) Glassman is widely used in towns along the Apulian coast, as it is partially resistant to marine aerosols, and particularly in Barletta where it characterizes one of the main streets of the city (Figure 7).

From the comparison made on the Portal to the flora of Italy 2024.2 https://dryades.units.it/floritaly/ (accessed on 15 June 2024) with other Italian regions, the following interesting results emerged*:* 111 taxa (38.5%) out of 288 taxa surveyed are new to Apulia, meaning by new taxa that those taxa were erroneously reported for Apulia previously but are now confirmed by us or other taxa that have so far escaped reporting because of poor exploration of the Apulian territory or because they have not yet begun the process of spontaneization. *Cedrus libani* A. Rich, *Phoenix dactylifera* L., *Photinia serratifolia* (Desf.) Kalkman cv*. Red Robin*, *Phyllostachys nigra* (Lodd. ex Lindl.) Munro, *Picea abies* (L.) H. Karst., *Pinus halepensis* Mill. subsp*. brutia* (Ten.) Holmboe, *Platanus* ×*hispanica* Mill. ex Münchh. *Portulacaria afra* Jacq. *Prunus cerasifera* Ehrh, and *Schinus terebinthifolia* Raddi are new reports for Apulia.

*Abies alba* Mill., *Berberis vulgaris* L., *Crataegus rhipidophylla* Gand., *Myoporum laetum* G. Forst., *Platanus orientalis* L., *Quercus petraea* (Matt.) Liebl., and *Solanum laciniatum* Aiton were indicated as erroneous or doubtful reports for Apulia in the Portal to the flora of Italy 2024.2 and were found by us in Apulian territory. A scattered distribution in Italy is noted for *Agave sisalana* Perrine (Apulia, Sicily, and Sardinia), *Asparagus africanus* Lam. (Tuscany and Apulia), *Polygala myrtifolia* L. (Apulia, Liguria, Sicily, and Sardinia), *Quercus trojana* Webb and *Syagrus romanzoffiana* (Cham.) Glassman (Apulia and Basilicata), *Prunus webbii* (Spach) Vierh (Apulia and Sicily), *Vachellia farnesiana* (L.) Wight and Arn. (Apulia, Calabria, Sicily, and Sardinia), and *Aloe arborescens* Mill. and *Cestrum parqui* (Lam.) L’Her. (distributed only in south Italy).

*Viburnum rhytidophyllum* Hemsl., previously reported only for northeast Italy, and *Pyrus calleryana* Decne, so far known only for Emilia-Romagna, represent new reports for Apulia that extend the distribution of these taxa to southern Italy as well.

Finally, it is confirmed that only in Apulia is there the presence of *Cereus repandus* (L.) Mill. and *Cistus ×purpureus* Lam.

## 3. Discussion

More recent botanical studies have neglected the investigation on ornamental plants despite the fact that many plants introduced within Botanical Gardens over the past centuries have subsequently spread throughout the Italian regions such as, for example, *Cedrus deodara* (Roxb. ex D. Don) G. Don, *Chrysojasminum fruticans* (L.) Banfi, *Citrus reticulata*, *Eriobotrya japonica* (Thunb.) Lindl, and *Parthenocissus quinquefolia* (L.) Planch, and some others have become spontaneous such as *Agave americana* L., *Robinia pseudoacacia* L., and *Ailanthus altissima* (Mill.) Swingle, coming to characterize the landscape of vast areas of Italian territory.

**Figure 8:**
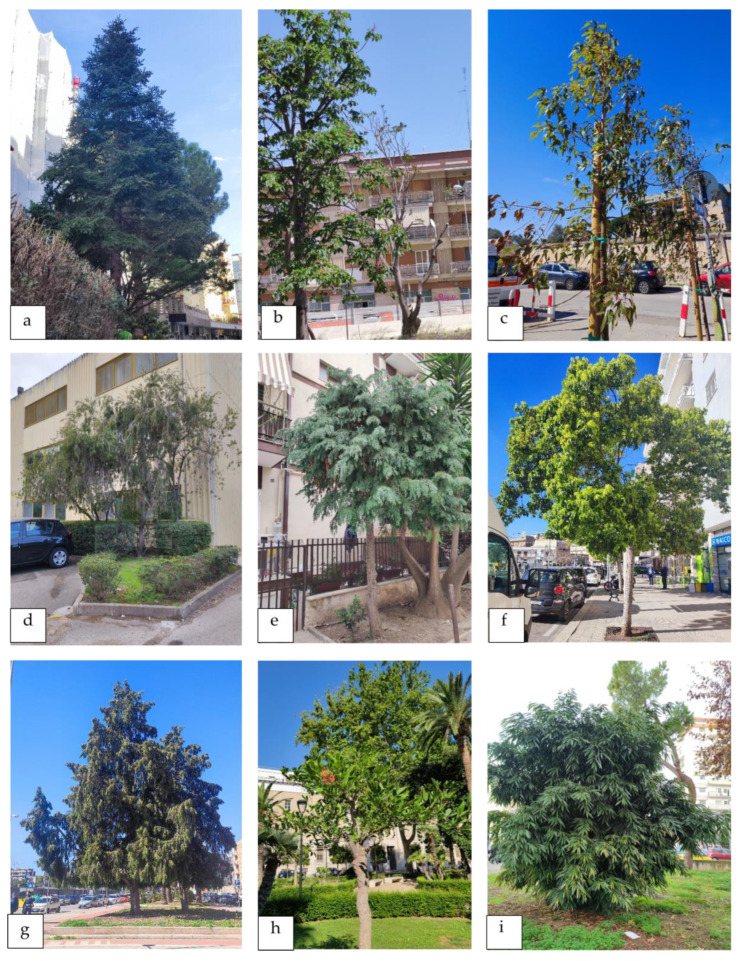
Ornamental plants surveyed in private gardens, hospitals, and streets of the city of Bari. (**a**) *Abies cephalonica*, (**b**) *Aesculus hippocastanum*, (**c**) *Brachychiton rupestris*, (**d**) *Melaleuca citrina*, (**e**) *Chamaecyparis lawsoniana*, (**f**) *Camphora officinarum*, (**g**) *Cupressus cashmeriana*, (**h**) *Erythrina crista-galli*, and (**i**) *Ficus maclellandii*.

**Figure 9:**
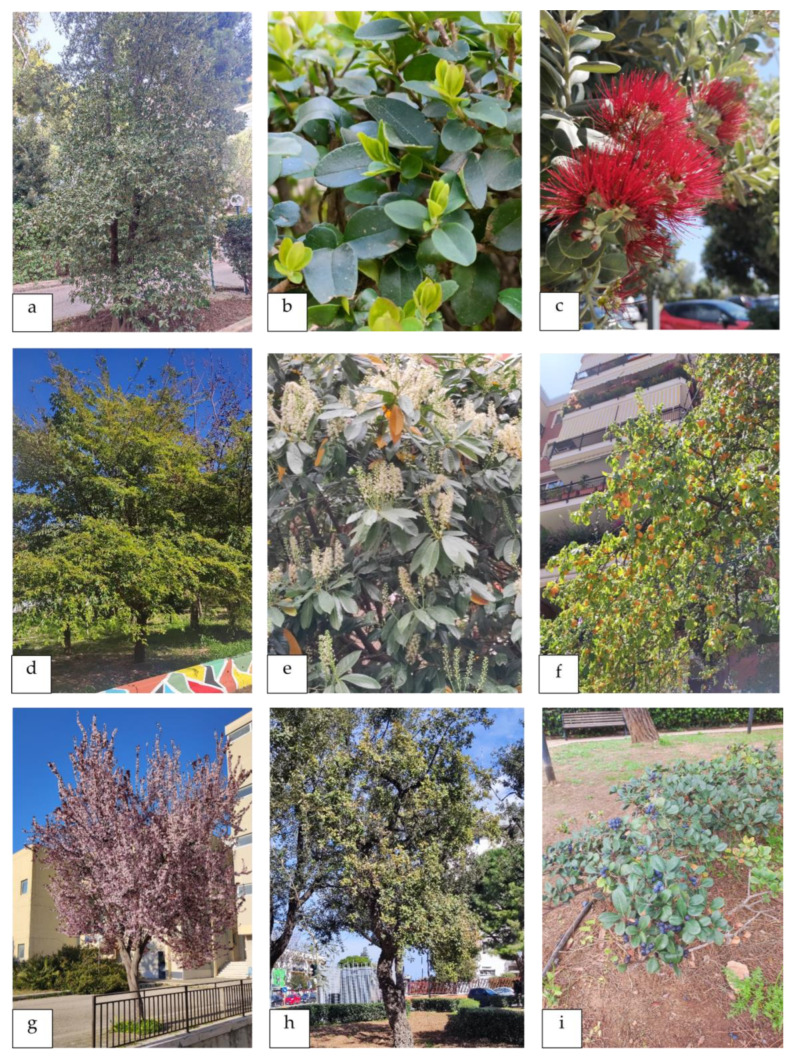
Ornamental plants surveyed in private gardens, hospitals, and streets of the city of Bari. (**a**) *Lagunaria patersonia*, (**b**) *Ligustrum ovalifolium*, (**c**) *Metrosideros excelsa*, (**d**) *Ulmus parvifolia*, (**e**) *Phytolacca dioica*, (**f**) *Prunus domestica*, (**g**) *Prunus cerasifera* subsp*. pissardii*, (**h**) *Quercus suber*, and (**i**) *Rhaphiolepis indica*.

Our study highlights the abundant presence of ornamental plants in comparison with native species (Figure 8 and Figure 9) not only within urban centers but throughout the region of Apulia. The checklist is an unpublished contribution to the knowledge of ornamental plants in an Italian southern region such as Apulia, which, due to its geographical location and land characteristics, hosts a rich contingent of ornamental species from different geographical origins.

The high percentage of alien species (more than 50%) detected among those surveyed is another important finding to assess the role of such species used as ornamentals, especially in cities. In fact, with the funding of the National Recovery and Resilience Plan (NRP), the focus on alien species is back on the agenda, and efforts are being made to raise awareness of the risks of the introduction and spread of invasive alien species in our country with a focus on the most correct practices, both in terms of production and gardening activities, to limit/contain the risk.

Apart from *Acacia saligna* (Labill.) H.L. Wendl., *Ailanthus altissima* (Mill.) Swingle, and *Robinia pseudoacacia* L., by now invasive in most of the Italian territory, among the taxa surveyed in this survey, there are no cases that would let us assume with certainty, to date, the transformation into invasive aliens. Some doubt is actually left by *Parkinsonia aculeata* L., a species rapidly expanding in the Apulian territory as highlighted by Pardi et al. [20] and is already reported as an invasive alien also in Sicily and Sardinia by the Portal to the flora of Italy.

The checklist of ornamental trees, shrubs, and succulents of Apulia becomes also a useful tool for municipal governments to direct future choices within cities with particular reference to the health of inhabitants. For example, many of the ornamental plants are found near schools and within public gardens frequented by children and the elderly, two of the categories most exposed to the growing problem of allergies.

The most well-known ornamental plants considered as responsible for pollinosis are *Cupressus* sp. pl., *Hesperocyparis* sp. pl.*, Pinus* sp. pl*., Olea europaea* L., *Quercus ilex* L., and *Populus* sp. pl., all plants that are widely used within Apulian cities. Also of wide use are some poisonous plants such as *Melia azedarach* L., *Nerium oleander* L., *Nicotiana glauca* Graham, and *Thuja occidentalis* L., as well as plants causing irritation to the eyes and hands due to the presence of latex in the leaves and stems (*Euphorbia* sp. pl. and *Ficus* sp. pl.). In addition, the wide use as ornamental of *Brachychiton* spp. and *Lagunaria patersonia* (Andrews) G. Don, due to the presence of stinging hairs inside the fruits, exposes citizens to damage on the mucous membranes of the eyes and mouth.

For what has been stated above, we can say that in most cases the choices made by the Administrations in term of ornamental plants are dictated by the aesthetics of the plants and the availability of them in nurseries rather than by a careful evaluation carried out with the professional support of botanists.

## 4. Materials and Methods

Periodic observations on the presence of ornamental trees, shrubs, and succulents in street trees, parks, and private and historic gardens of the six provinces of Apulia region, i.e., Bari, Barletta-Andria-Trani, Brindisi, Foggia, Lecce, and Taranto, have been carried out. Each province was visited twice in spring and autumn between 2021 and 2024. The names of the plants are listed alphabetically, and the presence within the province is indicated with an asterisk. The checklist comprises also hybrids and cultivars. The binomials and trinomials follow the database Plants of the World Online (https://powo.science.kew.org/) of the Kew Royal Botanical Gardens. The occurrence status of each taxon refers to Galasso et al. [17]. Recorded taxa are arranged in Appendix A Table A1 which comprises the binomial and trinomial, the family to which it belongs, and the presence/absence in the investigated provinces indicated, respectively, with an asterisk (★) and with a hyphen (-). An additional Appendix A Table A2 is present in which each census taxon is matched with its geographic origin, biological form, and alien or native species status. All of this information was obtained from the database Plants of the World Online (https://powo.science.kew.org/) of the Kew Royal Botanical Gardens. In Appendix A Table A1, in addition to species in sensu strictu, the forms, varieties, hybrids, and cultivars are included. Among the latter, as highlighted in Appendix A Table A2, artificial hybrids are also indicated as AH. The Portal to the flora of Italy 2024.2 https://dryades.units.it/floritaly/ (accessed on 15 June 2024) was used to compare the occurrence of the taxa we surveyed with other Italian regions. Taxa that are either new to Apulia in the above-mentioned Portal or erroneously reported for Apulia previously but confirmed by us, and other taxa that have so far escaped reporting due to the scarce exploration of the Apulian territory, or because they have not yet begun the process of spontaneization, have thus been marked with the symbol (●) in Appendix A Table A1.

## 5. Conclusions

In conclusion, our study makes a relevant contribution to the advancement of botanical studies in the Apulia region, being the first census on ornamental species. Even at the national level, such a type of census is so far very limited The data reported in this study are useful in noting the high rate of alien species in the Apulian territory resulting from often illogical choices made by municipal administrations that, without taking into account the possible problems related to biological invasions and the health of the most fragile citizens such as children and the elderly, leveraging only the material readily available in regional and extra-regional nurseries, they introduce species of all types and origins without consulting botanical experts. Our hope is that this census can be a useful basis for discussion with local authorities for a future public green policy based on rational choices also shared by citizens.

## Data Availability

The data that support the findings of this study are available upon request from the corresponding author.

## Appendix A

**Table A1:** List of ornamental plants in the six provinces of Apulia. BA = Bari; BAT = Barletta-Andria-Trani; BR = Brindisi; FG = Foggia; LE = Lecce; TA = Taranto. The presence/absence in the investigated provinces is indicated, respectively, with an asterisk (★) and with a hyphen (**-**). The symbol (●) is used to denote taxa that are either new to Apulia or erroneously reported for Apulia previously but confirmed by us and other taxa that have so far escaped reporting due to the scarce exploration of the Apulian territory or because they have not yet begun the process of spontaneization.

Taxa	Families	BA	BAT	BR	FG	LE	TA
*Abies alba* Mill.	Pinaceae	★	-	-	★	-	-
(●) *Abies cephalonica* Loudon	Pinaceae	★	-	★	-	-	-
*Acacia dealbata* Link	Fabaceae	★	★	★	★	★	★
*Acacia saligna* (Labill.) H.L. Wendl.	Fabaceae	★	★	★	★	★	★
*Acer campestre* L.	Sapindaceae	★	-	-	★	-	-
*Acer negundo* L.	Sapindaceae	★	-	-	★	-	★
*Aesculus hippocastanum* L.	Sapindaceae	★	-	-	★	-	★
(●) *Agapanthus africanus* (L.) Hoffmanns	Amaryllidaceae	★	★	★	★	★	★
*Agave americana* L.	Asparagaceae	★	★	★	★	★	★
(●) *Agave americana* L. f. *marginata*	Asparagaceae	★	★	★	★	★	★
*Agave sisalana* Perrine	Asparagaceae	★	-	-	-	-	-
*Ailanthus altissima* (Mill.) Swingle	Simaroubaceae	★	★	★	★	★	★
(●) *Allocasuarina torulosa* (Aiton) L.A.S. Johnson	Casuarinaceae	★	★	★	★	★	★
(●) *Alocasia macrorrhizos* (L.) G. Don	Araceae	★	-	-	-	-	-
*Aloe arborescens* Mill.	Asphodelaceae	★	★	★	★	★	★
(●) *Aloe vera* (L.) Burm. f.	Asphodelaceae	★	★	★	★	★	-
(●) *Annona cherimola* Mill.	Annonaceae	★	-	-	-	-	-
*Anthyllis barba-jovis* L.	Fabaceae	★	-	-	-	-	-
(●) *Araucaria columnaris* (G. Forst.) Hook.	Araucariaceae	★	-	-	-	-	-
(●) *Araucaria heterophylla* (Salisb.) Franco	Araucariaceae	★	★	★	★	★	★
*Arbutus unedo* L.	Ericaceae	★	★	★	★	★	★
*Asparagus acutifolius* L.	Asparagaceae	★	-	-	-	-	-
*Asparagus aethiopicus* L.	Asparagaceae	★	-	-	-	-	-
*Asparagus africanus* Lam.	Asparagaceae	★	-	-	-	-	-
(●) *Asparagus asparagoides* (L.) Druce	Asparagaceae	★	-	-	-	-	-
(●) *Asparagus densiflorus* (Kunth) Jessop	Asparagaceae	★	★	★	★	★	★
*Atriplex halimus* L.	Amaranthaceae	★	★	★	★	★	★
*Austrocylindropuntia subulata* (Muehlenpf.) Backeb	Cactaceae	-	★	-	★	-	-
(●) *Bambusa vulgaris* Schrad. ex J.C. Wendl.	Poaceae	★	★	★	★	★	★
(●) *Bauhinia variegata* L. var*. candida* Voigt	Fabaceae	★	-	-	-	-	-
*Berberis aquifolium* Pursh	Berberidaceae	★	-	-	-	-	-
(●) *Berberis thunbergii* DC. f. *atropurpuea*	Berberidaceae	★	-	-	★	-	★
*Berberis vulgaris* L.	Berberidaceae	★	-	-	-	-	-
(●) *Bougainvillea glabra* Choisy	Nyctaginaceae	★	★	★	★	★	★
(●) *Bougainvillea spectabilis* Willd.	Nyctaginaceae	★	★	★	★	★	★
(●) *Brachychiton acerifolius* (A.Cunn. ex G. Don) F. Muell.	Malvaceae	-	-	-	-	★	-
(●) *Brachychiton discolor* F. Muell.	Malvaceae	★	-	-	-	-	-
(●) *Brachychiton diversifolius* R. Br.	Malvaceae	★	-	-	-	-	-
(●) *Brachychiton rupestris* (T. Mitch. ex Lindl.) K. Schum.	Malvaceae	★	-	-	-	★	-
(●) *Brahea armata* S. Watson	Arecaceae	★	-	-	-	-	-
*Broussonetia papyrifera* (L.) L’Her. ex Vent.	Moraceae	★	-	-	-	★	-
(●) *Brugmansia arborea* (L.) Sweet.	Solanaceae	★	★	★	-	★	★
*Butia capitata* (Mart.) Becc	Arecaceae	★	-	★	-	-	★
*Buxus sempervirens* L.	Buxaceae	★	★	★	★	★	★
(●) *Calocedrus decurrens* (Torr.) Florin f. *aureovariegata*	Cupressaceae	★	-	-	-	-	-
(●) *Camphora officinarum* Boerh. ex Fabr.	Lauraceae	★	★	-	-	★	-
*Campsis radicans* (L.) Bureau.	Bignoniaceae	★	★	★	★	★	★
*Capparis spinosa* Desf. var. *ovata* (Desf.) Sm.	Capparaceae	★	★	★	★	★	★
*Carpinus orientalis* Mill.	Betulaceae	★	-	-	★	-	-
(●) *Carissa macrocarpa* (Eckl.) A. DC.	Apocynaceae	★	★	★	★	★	★
(●) *Caryota urens* L.	Arecaceae	★	-	-	-	★	-
*Casuarina equisetifolia* L.	Casuarinaceae	★	★	★	★	★	★
(●) *Catalpa bignonioides* Walter	Bignoniaceae	★	★	★	-	★	-
*Cedrus atlantica* (Endl.) Manetti ex Carrière	Pinaceae	★	★	★	★	★	★
(●) *Cedrus deodara* (Roxb. ex D. Don) G. Don	Pinaceae	★	★	★	★	★	★
*Cedrus libani* A. Rich.	Pinaceae	★	-	-	★	★	-
(●) *Ceiba speciosa* (A. St.-Hil., A. Juss. and Cambess.) Ravenna	Malvaceae	★	-	★	-	★	-
*Celtis australis* L.	Cannabaceae	★	★	★	★	★	★
(●) *Celtis occidentalis* L.	Cannabaceae	-	-	-	-	-	★
*Ceratonia siliqua* L.	Fabaceae	★	★	★	★	★	★
*Cercis siliquastrum* L.	Fabaceae	★	★	★	★	★	★
*Cereus repandus* (L.) Mill.	Cactaceae	★	-	-	-	-	-
*Cestrum parqui* (Lam.) L’Her.	Solanaceae	★	-	-	-	★	-
(●) *Chamaecyparis lawsoniana* (A. Murray bis) Parl.	Cupressaceae	★	-	-	-	-	-
*Chamaerops humilis* L.	Arecaceae	★	★	★	★	★	★
(●) *Chrysojasminum fruticans* (L.) Banfi	Oleaceae	★	-	-	-	-	-
*Cistus × purpureus* Lam.	Cistaceae	★	-	-	-	-	-
*Citrus × limon* (L.) Osbeck	Rutaceae	★	★	★	★	★	★
(●) *Citrus polytrifolia* Govaerts	Rutaceae	★	-	-	-	-	-
(●) *Citrus reticulata* Blanco	Rutaceae	★	-	-	★	-	-
(●) *Citrus ×sinensis* (L.) Osbeck	Rutaceae	★	-	-	-	-	-
(●) *Cordyline australis* (G. Forst.) Endl.	Asparagaceae	★	★	★	★	★	★
*Cornus sanguinea* L.	Cornaceae	★	-	-	-	-	-
*Cortaderia selloana* (Schult. and Schult. f.) Asch. and Graebn.	Poaceae	★	★	★	-	★	-
*Corylus avellana* L.	Betulaceae	★	-	-	-	-	-
(●) *Cotoneaster divaricatus* Rehder and E.H. Wilson	Rosaceae	★	-	-	★	-	-
*Crataegus rhipidophylla* Gand.	Rosaceae	★	-	-	-	-	-
(●) *Cupressus cashmeriana* Royle ex Carriere	Cupressaceae	★	-	-	-	-	-
*Cupressus sempervirens* L. var. *horizontalis* (Mill.) Loudon	Cupressaceae	★	-	-	★	★	★
*Cupressus sempervirens* L.	Cupressaceae	★	★	★	★	★	★
(●) *Cycas revoluta* Thunb.	Cycadaceae	★	★	★	★	★	★
*Cydonia oblonga* Mill.	Rosaceae	★	-	-	-	-	-
(●) *Cyperus papyrus* L.	Cyperaceae	★	★	★	★	★	★
(●) *Dasylirion serratifolium* (Karw. ex Schult. and Schult. f.) Zucc.	Asparagaceae	-	★	-	-	-	-
*Dendropanax arboreus* (L.) Decne and Planch. f. *variegata*	Araliaceae	★	★	★	-	★	-
(●) *Deutzia gracilis* Siebold and Zucc.	Hydrangeaceae	★	-	-	-	-	-
(●) *Deutzia scabra* Thunb.	Hydrangeaceae	★	-	-	-	-	-
(●) *Dolichandra unguis-cati* (L.) L.G. Lohmann	Bignoniaceae	★	-	★	-	★	-
*Duranta erecta* L.	Verbenaceae	★					
(●) *Elaeagnus × ebbingei* J. Door.	Elaeagnaceae	★	★	★	★	★	★
(●) *Elaeagnus × submacrophylla* Servett.	Elaeagnaceae	★	★	★	★	★	★
*Eriobotrya japonica* (Thunb.) Lindl.	Rosaceae	★	★	★	★	★	★
(●)*Erythrina crista-galli* L.	Fabaceae	★	-	-	-	-	-
*Erythrina herbacea* L.	Fabaceae	-	-	-	-	-	★
*Erythrostemon gilliesii* (Hook.) Klotzsch	Fabaceae	★	-	-	★	-	-
*Eucalyptus camaldulensis* Dehnh.	Myrtaceae	★	★	★	★	★	★
*Eugenia uniflora* L.	Myrtaceae	★	★	★	-	★	-
*Euonymus japonicus* Thunb.	Celastraceae	★	★	★	★	★	★
(●) *Euonymus japonicus* Thunb. f. *aureo-variegatus*	Celastraceae	★	★	★	★	★	-
(●) *Euonymus pulchellus* Jacob-Makoj	Celastraceae	★	-	-	-	-	-
*Euphorbia dendroides* L.	Euphorbiaceae	★	-	-	-	-	-
*Euphorbia ingens* E. May. ex Boiss.	Euphorbiaceae	★	-	-	-	-	-
(●) *Euphorbia murielii* N.E.Br.	Euphorbiaceae	-	-	-	-	-	★
*Euphorbia tirucalli* L.	Euphorbiaceae	-	-	-	-	★	-
(●) *Euryops pectinatus* (L.) Cass.	Asteraceae	★	-	-	-	-	-
*Fallopia baldeschuanica* (Regel) Holub	Polygonaceae	★	-	-	★	-	-
(●) *Feijoa sellowiana* (O. Berg) O. Berg.	Myrtaceae	★	-	-	-	★	-
*Ficus carica* L.	Moraceae	★	★	★	★	★	★
(●) *Ficus elastica* Roxb. ex Hornem.	Moraceae	★	-	-	-	★	-
(●) *Ficus elastica* Hornem. var. *decora* Guillaumin	Moraceae	★	★	★	-	-	-
*Ficus maclellandii* King	Moraceae	★	-	-	-	-	-
(●) *Ficus rubiginosa* Desf.	Moraceae	★	-	-	-	-	-
(●) *Firmiana simplex* (L.) W. Wight	Malvaceae	★	-	-	-	-	-
(●) *Fraxinus angustifolia* subsp. *oxycarpa* (M. Bieb. ex Willd.) Franco and Rocha Afonso	Oleaceae	★	-	-	★	-	-
*Fraxinus excelsior* L.	Oleaceae	★	-	-	★	-	-
*Fraxinus ornus* L.	Oleaceae	★	-	-	★	-	★
(●) *Furcraea selloana* K. Koch	Asparagaceae	-	-	★	-	-	-
(●) *Ginkgo biloba* L.	Ginkgoaceae	★	-	-	★	-	★
*Gleditsia triacanthos* L.	Fabaceae	★	-	-	-	-	-
(●) *Grevillea robusta* A. Cunn. ex R. Br.	Proteaceae	★	-	★	-	★	-
*Hedera helix* L.	Araliaceae	★	★	★	★	★	★
*Hedera canariensis* Willd.	Araliaceae	★	★	★	★	★	★
*Hesperocyparis harizonica* (Greene) Bartel	Cupressaceae	★	★	★	★	★	★
(●) *Hesperocyparis macrocarpa* (Hartw.) Bartel.	Cupressaceae	★	★	★	★	★	★
(●)×*Hesperotropsis leylandii* (A.B. Jachs- and Dallim.) Garl. and Jerry Moore	Cupressaceae	★	★	★	★	★	★
(●) *Hibiscus moscheutos* L.	Malvaceae	-	-	-	-	-	★
(●) *Hibiscus × rosa-sinensis* L.	Malvaceae	★	★	★	-	★	-
*Hibiscus syriacus* L.	Malvaceae	★	★	★	★	-	★
*Ilex aquifolium* L.	Aquifoliaceae	★	-	-	★	-	-
(●) *Jacaranda mimosifolia* D. Don	Bignoniaceae	★	★	★	★	★	★
*Jacobaea maritima* (L.) Pelser and Meijden	Asteraceae	★	★	★	★	★	★
(●) *Jasminum officinale* L.	Oleaceae	★	-	-	-	-	-
(●) *Jasminum polyanthum* Franch.	Oleaceae	★	★	★	★	★	★
*Juglans regia* L.	Juglandaceae	★	★	★	★	★	★
*Juniperus chinensis* L. f. *Pfitzeriana Glauca*	Cupressaceae	★	★	★	★	★	★
*Juniperus horizontalis* Moench	Cupressaceae	★	★	-	★	★	★
*Juniperus oxycedrus* L.	Cupressaceae	★	-	-	-	-	-
(●) *Justicia adhatoda* L.	Acanthaceae	★	-	-	-	-	-
*Koelreuteria paniculata* Laxm.	Sapindaceae	★	-	★	-	★	-
(●) *Lagerstroemia indica* L.	Lythraceae	★	★	★	★	★	★
(●) *Lagunaria patersonia* (Andrews) G .Don	Malvaceae	★	-	-	-	-	★
*Lantana camara* L.	Verbenaceae	★	★	★	★	★	★
(●) *Lantana montevidensis* (Spreng.) Brig.	Verbenaceae	★	★	★	-	★	-
*Laurus nobilis* L.	Lauraceae	★	★	★	★	★	★
*Lavandula angustifolia* Mill.	Lamiaceae	★	★	-	-	-	-
*Ligustrum japonicum* Thunb.	Oleaceae	★	★	★	★	★	★
*Ligustrum japonicum* Thunb. f. *aureo-variegatum*	Oleaceae	★	-	★	★	★	★
*Ligustrum lucidum* W.T. Aiton	Oleaceae	★	★	★	★	★	★
*Ligustrum ovalifolium* Hassk.	Oleaceae	★	-	-	-	-	-
(●) *Ligustrum sinense* Lour.	Oleaceae	★	-	-	-	-	-
(●) *Liquidambar styraciflua* L.	Altingiaceae	★	-	-	-	-	★
(●) *Livistona chinensis* (Jacq.) R. Br. Ex Mart..	Arecaceae	★	-	-	-	-	-
*Lonicera caprifolium* L.	Caprifoliaceae	★					
(●) *Loropetalum chinense* (R. Br) Oliv. f. *rubrum* H.T. Chang	Hamamelidaceae	★	-	-	★	-	-
(●) *Magnolia grandiflora* L.	Magnoliaceae	★	★	★	★	★	★
(●) *Mandevilla laxa* (Ruiz and Pav.) Woodson	Apocynaceae	-	-	-	-	-	★
(●) *Melaleuca citrina* (Curtis) Dum. Cours.	Myrtaceae	★	★	★	★	★	★
*Melia azedarach* L.	Meliaceae	★	★	★	★	★	★
(●) *Metrosideros excelsa* Sol. ex Gaertn.	Myrtaceae	★	★	★	★	★	★
(●) *Morus alba* L. f. *pendula*	Moraceae	★	★	★	★	★	★
(●) *Murraya paniculata* (L.) Jack	Rutaceae	★	-	-	-	-	-
(●) *Musa × paradisiaca* L.	Musaceae	★	-	-	★	-	★
*Myoporum laetum* G. Forst.	Scrophulariaceae	-	-	-	-	-	★
*Myrtus communis* L.	Myrtaceae	★	★	★	★	★	★
*Myrtus communis* L. f. *Tarentina*	Myrtaceae	★	-	-	-	★	-
*Nandina domestica* Thunb.	Berberidaceae	★	★	★	★	★	★
*Nerium oleander* L.	Apocynaceae	★	★	★	★	★	★
*Nicotiana glauca* Graham.	Solanaceae	-	-	-	-	-	★
*Oenothera lindheimeri* (Engelm. and A. Gray) W.L. Wagner and Hook	Onagraceaea	★	-	-	-	-	-
*Olea europaea* L.	Oleaceae	★	★	★	★	★	★
*Opuntia ficus-indica* (L.) Mill.	Cactaceae	★	★	★	★	★	★
(●) *Osmanthus xburkwoodii* (Burkwood and Skipwith) P.S. Green	Oleaceae	★	-	-	-	-	-
*Ostrya carpinifolia* Scop.	Betulaceae	★	-	-	★	-	-
(●) *Paeonia* × *suffruticosa* Andrews	Paeoniaceae	★	-	-	-	-	-
*Parkinsonia aculeata* L.	Fabaceae	★	-	-	-	-	-
*Parthenocissus tricuspidata* (Siebold and Zucc.) Planch.	Vitaceae	★	-	-	-	-	-
*Parthenocissus quinquefolia* (L.) Planch.	Vitaceae	-	★	★	★	★	★
*Passiflora caerulea* L.	Passifloraceae	★	★	★	★	★	★
(●) *Persea americana* Mill.	Lauraceae	★	-	-	-	-	-
(●) *Persea indica* (L.) Spreng.	Lauraceae	★	-	-	-	-	-
*Petunia × atkinsiana* (Sweet) D. Don ex W.H. Baxter	Solanaceae	★	★	★	★	★	★
*Philadelphus inodorus* L.	Hydrangeaceae	★	★	★	★	★	★
*Phlomis fruticosa* L.	Lamiaceae	★	-	-	-	-	-
(●) *Phlomis purpurea* L.	Lamiaceae	★	-	-	-	-	-
*Phlomis viscosa* Poir.	Lamiaceae	★	-	-	-	-	-
*Phoenix canariensis* H. Wildpret	Arecaceae	★	★	★	★	★	★
*Phoenix dactylifera* L.	Arecaceae	★	★	★	★	★	★
*Photinia serratifolia* (Desf.) Kalkman cv*. Red Robin*	Rosaceae	★	★	★	★	★	★
*Phyllostachys aurea* (Andrè) Rivière and C. Rivière	Poaceae	★	-	-	-	-	-
*Phyllostachys nigra* (Lodd. ex Lindl.) Munro	Poaceae	★	★	★	-	★	-
*Phytolacca dioica* L.	Phytolaccaceae	★	★	★	★	★	★
*Picea abies* (L.) H. Karst.	Pinaceae	★	-	-	★	-	-
(●) *Picea pungens* Engelm. Cv. *Kosteriana*	Pinaceae	★	-	-	★	-	-
*Pinus canariensis* C. Sm. ex DC.	Pinaceae	★	★	★	-	★	-
*Pinus halepensis* Mill. subsp*. brutia* (Ten.) Holmboe	Pinaceae	★	-	-	-	-	-
*Pinus halepensis* Mill.	Pinaceae	★	★	★	★	★	★
*Pinus nigra* J. F. Arnold	Pinaceae	★	-	-	-	-	★
*Pinus pinea* L.	Pinaceae	★	★	★	★	★	★
*Pinus pinaster* Aiton	Pinaceae	★	★	-	-	-	-
*Pistacia lentiscus* L.	Anacardiaceae	★	★	★	-	★	-
*Pistacia terebinthus* L.	Anacardiaceae	★	★	★	-	★	-
*Pittosporum tobira* (Thunb.) W.T. Aiton	Pittosporaceae	★	★	★	★	★	★
*Pittosporum tobira* (Thunb.) W.T. Aiton f. *nanum*	Pittosporaceae	★	-	-	-	-	-
*Platanus × hispanica* Mill. ex Münchh.	Platanaceae	★	★	★	★	★	★
*Platanus orientalis* L.	Platanaceae	★	-	-	-	-	★
*Platycladus orientalis* (L.) Franco	Cupressaceae	★	★	★	★	★	★
*Plumbago auriculata* Lam.	Plumbaginaceae	★	★	★	★	★	★
*Polygala myrtifolia* L.	Polygalaceae	★	★	★	★	★	★
*Populus alba* L.	Salicaceae	★	★	★	★	★	-
*Populus nigra* L.	Salicaceae	★	★	★	★	★	★
*Portulacaria afra* Jacq.	Didiereaceae	★	★	★	-	★	-
*Prunus cerasifera* Ehrh.	Rosaceae	★	-	-	-	-	-
(●) *Prunus cerasifera* subsp*. pissardii* (Carrière) Dostál	Rosaceae	★	★	★	★	★	★
*Prunus domestica* L.	Rosaceae	★	-	-	-	-	★
*Prunus dulcis* (Mill.) D.A. Webb	Rosaceae	★	-	-	-	-	-
*Prunus laurocerasus* L.	Rosaceae	★	★	★	★	★	★
*Prunus mahaleb* L.	Rosaceae	★	-	-	-	-	-
*Prunus webbii* (Spach) Vierh.	Rosaceae	★	-	-	-	-	-
(●) *Pterocarya fraxinifolia* (Poir.) Spach	Juglandaceae	★	-	-	-	-	-
*Punica granatum* L.	Lythraceae	★	★	★	★	★	★
*Pyracantha coccinea* M. Roem.	Rosaceae	★	★	★	★	★	★
(●) *Pyrus calleryana* Decne.	Rosaceae	★	-	-	-	-	★
*Pyrus spinosa* Forssk.	Rosaceae	★	-	-	-	-	-
*Quercus cerris* L.	Fagaceae	★	-	-	-	-	-
*Quercus ilex* L.	Fagaceae	★	★	★	★	★	★
*Quercus ithaburensis* subsp*. macrolepis* (Kotschy) Hedge and Yalt.	Fagaceae	★	-	★	-	★	-
*Quercus petraea* (Matt.) Liebl.	Fagaceae	★	-	-	-	-	-
*Quercus pubescens* Willd.	Fagaceae	★	-	-	★	-	-
*Quercus robur* L.	Fagaceae	★	-	-	-	-	-
*Quercus suber* L.	Fagaceae	★	-	-	-	-	-
*Quercus trojana* Webb	Fagaceae	★	-	-	-	-	-
*Rhamnus alaternus* L.	Rhamnaceae	★	★	★	-	★	-
(●) *Rhaphiolepis indica* (L.) Lindl.	Rosaceae	★	-	-	-	-	-
(●) *Rhododendron ferrugineum* L.	Ericaceae	★	-	-	-	-	-
*Robinia pseudoacacia* L.	Fabaceae	★	★	★	★	★	★
*Rosa canina* L.	Rosaceae	★	-	-	-	-	-
*Rosa × hybrida* Vill. cv. *La Sevillana*	Rosaceae	★	★	★	★	★	-
*Ruscus aculeatus* L.	Asparagaceae	★	★	★	-	★	-
(●) *Sabal palmetto* (Walter) Lodd. ex Schult. and Schult.f.	Arecaceae	★	-	-	-	-	-
(●) *Salix babylonica* L.	Salicaceae	★	★	★	-	★	-
*Salvia fruticosa* Mill.	Lamiaceae	-	★	★	-	★	-
(●) *Salvia greggii* A.Gray	Lamiaceae	-	★	-	-	-	-
*Salvia officinalis* L.	Lamiaceae	★	★	★	-	★	-
*Salvia rosmarinus* Spenn.	Lamiaceae	★	★	★	★	★	★
*Schinus molle* L.	Anacardiaceae	★	★	★	★	★	★
*Schinus terebinthifolia* Raddi	Anacardiaceae	★	-	-	-	-	-
*Senecio angulatus* L. f.	Asteraceae	★	-	-	-	-	-
(●) *Senecio grandiflorus* P.J. Bergius	Asteraceae	★	-	-	-	-	-
(●) *Sideroxylon spinosum* L.	Sapotaceae	★	-	-	-	-	-
*Solanum laciniatum* Aiton	Solanaceae	-	★	-	-	-	-
*Spartium junceum* L.	Fabaceae	★	-	★	-	★	-
(●) *Sterlizia nicolai* Regel and Körn.	Strelitziaceae	★	★	★	★	★	★
(●) *Sterlizia reginae* Banks	Strelitziaceae	★	★	★	★	★	★
*Styphnolobium japonicum* (L.) Schott	Fabaceae	★	-	-	-	-	-
*Syagrus romanzoffiana* (Cham.) Glassman	Arecaceae	★	★	★	★	★	★
*Tamarix africana* Poir. var*. fluminensis* (Maire) Baum	Tamaricaceae	-	-	-	-	-	★
*Tamarix arborea* (Ehrenb.) Bunge var. *arborea*	Tamaricaceae	★	★	★	★	★	★
*Tamarix hampeana* Boiss. and Heldr.	Tamaricaceae	-	-	-	-	★	-
*Tamarix macrocarpa* Bunge	Tamaricaceae	-	-	-	★	-	-
*Tamarix meyeri* Boiss.	Tamaricaceae	★	-	-	-	★	-
*Tamarix parviflora* DC.	Tamaricaceae	★	★	★	★	★	★
*Tamarix rosea* Bunge	Tamaricaceae	-	★	-	-	-	★
(●) *Tecoma capensis* Lindl.	Bignoniaceae	★	★	★	★	★	★
*Teucrium fruticans* L.	Lamiaceae	-	★	★	-	★	-
*Thuja occidentalis* L.	Cupressaceae	★	★	★	★	★	★
(●) *Tilia americana* L.	Malvaceae	★	-	-	-	-	-
*Tilia cordata* Mill.	Malvaceae	★	★	★	★	★	★
*Tilia platyphyllos* Scop.	Malvaceae	★	-	-	★	-	-
*Tilia × europaea* L.	Malvaceae	★	-	-	-	-	-
(●) *Trachelospermum jasminoides* Lem.	Apocynaceae	★	★	★	★	★	★
(●) *Trachycarpus fortunei* (Hook) Wendl	Arecaceae	★	★	★	★	★	★
(●) *Trema micranthum* (L.) Blume	Cannabaceae	★	-	-	-	-	-
*Ulmus laevis* Pall.	Ulmaceae	-	★	-	★	-	-
*Ulmus minor* Mill.	Ulmaceae	★	★	★	★	★	-
*Ulmus minor* Mill. subsp. *canescens* Bartolucci and Galasso	Ulmaceae	★	-	-	-	-	-
(●) *Ulmus parvifolia* Jacq.	Ulmaceae	★	-	-	-	-	-
*Ulmus pumila* L.	Ulmaceae	-	-	★	★	-	-
(●) *Vachellia farnesiana* (L.) Wight and Arn.	Fabaceae	★	-	-	-	-	-
*Vachellia karroo* (Hayne) Banfi and Galasso	Fabaceae	★	★	★	★	★	★
*Viburnum lucidum* Mill.	Viburnaceae	★	★	★	★	★	★
(●) *Viburnum rhytidophyllum* Hemsl.	Viburnaceae	★	-	-	★	-	-
*Viburnum tinus* L.	Viburnaceae	★	★	★	★	★	★
*Vitex agnus-castus* L.	Lamiaceae	★	★	★	★	★	★
*Washingtonia filifera* (T. Moore and Mast.) H. Wendl. ex de Bary	Arecaceae	★	★	★	★	★	★
*Washingtonia robusta* H. Wendl.	Arecaceae	★	★	★	★	★	★
*Wisteria sinensis* (Sims) DC:	Fabaceae	★	★	★	★	★	★
(●) *Yucca aloifolia* L.	Asparagaceae	★	★	★	★	★	★
*Yucca gigantea* Lem	Asparagaceae	★	★	★	★	★	★
*Yucca gloriosa* L.	Asparagaceae	★	-	-	-	-	-
(●) *Yucca rostrata* Engelm. ex Trel.	Asparagaceae	★	-	★	-	-	-

**Table A2:** List of ornamental plants per geographical origin (GO), growth form (GF), and native (N), Alien (A), and Artificial Hybrid (AH) status.

Taxa	GO	Growth Form	Native/Alien
*Abies alba* Mill.	Europe/Balkans	P scap	N
*Abies cephalonica* Loudon	Greece	P scap	A
*Acacia dealbata* Link	Australia	P scap	A
*Acacia saligna* (Labill.) H.L. Wendl.	Australia	P scap	A
*Acer campestre* L.	Europe/Caucasus/N.W. Africa	P scap	N
*Acer negundo* L.	C.America	P scap	A
*Aesculus hippocastanum* L.	Balkans/Caucasus	P scap	A
*Agapanthus africanus* (L.) Hoffmanns	South Africa	G rhiz	A
*Agave americana* L.	C. America	P caesp	A
*Agave americana* L. f. *marginata*	C. America	P caesp	A
*Agave sisalana* Perrine	C. America	P caesp	A
*Ailanthus altissima* (Mill.) Swingle	S.E. Asia	P scap	A
*Allocasuarina torulosa* (Aiton) L.A.S. Johnson	Australia	P scap	A
*Alocasia macrorrhizos* (L.) G. Don	Australia/S.E. Asia	G rhiz	A
*Aloe arborescens* Mill.	S.E. Africa	NP	A
*Aloe vera* (L.) Burm. f.	South Arabia	NP	A
*Annona cherimola* Mill.	W.S. America	P caesp/P.scap	A
*Anthyllis barba-jovis* L.	W.C. Medit	P caesp	N
*Araucaria columnaris* (G. Forst.) Hook.	Australia	P scap	A
*Araucaria heterophylla* (Salisb.) Franco	Australia	P scap	A
*Arbutus unedo* L.	Medit.	P caesp/P.scap	N
*Asparagus acutifolius* L.	Medit.	G rhiz/NP	N
*Asparagus aethiopicus* L.	South Africa	G rhiz/NP	A
*Asparagus africanus* Lam.	South Africa/Arabia/W.India	G rhiz	A
*Asparagus asparagoides* (L.) Druce	E.S. Africa	G rhiz	A
*Asparagus densiflorus* (Kunth) Jessop	E.S. Africa	G rhiz	A
*Atriplex halimus* L.	Macaronesia	P caesp	N
*Austrocylindropuntia subulata* (Muehlenpf.) Backeb	S. America	H scap	A
*Bambusa vulgaris* Schrad. ex J.C. Wendl.	S.E. Asia	P scap	A
*Bauhinia variegata* L. var*. candida* Voigt	S.E. Asia	P scap	A
*Berberis aquifolium* Pursh	N.C. America	P caesp	A
*Berberis thunbergii* DC. f. *atropurpuea*	Japan	P caesp	A
*Berberis vulgaris* L.	Europe/Caucasus	P caesp	A
*Bougainvillea glabra* Choisy	S. America	P lian	A
*Bougainvillea spectabilis* Willd.	S. America	P lian	A
*Brachychiton acerifolius* (A.Cunn. ex G. Don) F. Muell.	E. Australia	P scap	A
*Brachychiton discolor* F. Muell.	E. Australia	P scap	A
*Brachychiton diversifolius* R. Br.	N.W. Australia	P scap	A
*Brachychiton rupestris* (T. Mitch. ex Lindl.) K. Schum.	N.E. Australia	P scap	A
*Brahea armata* S. Watson	C. America	P scap	A
*Broussonetia papyrifera* (L.) L’Her. ex Vent.	S.E. Asia	P caesp/P scap	A
*Brugmansia arborea* (L.) Sweet.	W.S. America	P scap	A
*Butia capitata* (Mart.) Becc	S.W. America	P scap	A
*Buxus sempervirens* L.	Europe/Caucasus/N. Africa	P caesp/P scap	N
*Calocedrus decurrens* (Torr.) Florin f. *aureovariegata*	C. W. America	P scap	A
*Camphora officinarum* Boerh. ex Fabr.	E. Asia	P scap	A
*Campsis radicans* (L.) Bureau.	C. E. America	P lian	A
*Capparis spinosa* Desf. var. *ovata* (Desf.) Sm.	Sicily/N. Africa	NP	N
*Carpinus orientalis* Mill.	S.E. Europe/Caucasus	P scap	N
*Carissa macrocarpa* (Eckl.) A. DC.	C.S. Africa	P caesp	A
*Caryota urens* L.	India	P scap	A
*Casuarina equisetifolia* L.	S.E. Asia/Australia	P scap	A
*Catalpa bignonioides* Walter	C.E. America	P scap	A
*Cedrus atlantica* (Endl.) Manetti ex Carrière	N.W. Africa	P scap	A
*Cedrus deodara* (Roxb. ex D. Don) G. Don	W. Asia	P scap	A
*Cedrus libani* A. Rich.	Lebanon/Caucasus	P scap	A
*Ceiba speciosa* (A. St.-Hil., A. Juss. and Cambess.) Ravenna	S.America	P scap	A
*Celtis australis* L.	S. Europe/Caucasus/N.W.Africa	P scap	A
*Celtis occidentalis* L.	N.C. America	P scap	A
*Ceratonia siliqua* L.	S. Europe/Caucasus/N.W. Africa	P scap	N
*Cercis siliquastrum* L.	S.W: Europe/Caucasus	P scap	N
*Cereus repandus* (L.) Mill.	N.W. South America	Ch succ	A
*Cestrum parqui* (Lam.) L’Her.	S. America	NP	A
*Chamaecyparis lawsoniana* (A. Murray bis) Parl.	C. W. America	P scap	A
*Chamaerops humilis* L.	Medit./N. Africa	P caesp	N
*Chrysojasminum fruticans* (L.) Banfi	S. Europe/Caucasus/N.W. Africa	P caesp	N
*Cistus × purpureus* Lam.	AH	P caesp/P scap	AH
*Citrus × limon* (L.) Osbeck	AH	P scap	AH
*Citrus polytrifolia* Govaerts	China	P caesp/P scap	A
*Citrus reticulata* Blanco	China	P caesp/P scap	A
*Citrus × sinensis* (L.) Osbeck	AH	P scap	AH
*Cordyline australis* (G. Forst.) Endl.	New Zealand	P scap	A
*Cornus sanguinea* L.	Europe/Caucasus	P caesp/P scap	N
*Cortaderia selloana* (Schult. and Schult. f.) Asch. and Graebn.	S. America	H caesp	A
*Corylus avellana* L.	Europe/Caucasus	P caesp/P scap	N
*Cotoneaster divaricatus* Rehder and E.H. Wilson	China	P caesp	A
*Crataegus rhipidophylla* Gand.	Europe/Caucasus	P scap	A
*Cupressus cashmeriana* Royle ex Carriere	India	P scap	A
*Cupressus sempervirens* L. var. *horizontalis* (Mill.) Loudon	Caucasus/C.N. Africa	P scap	A
*Cupressus sempervirens* L.	Caucasus/C.N. Africa	P scap	A
*Cycas revoluta* Thunb.	China/Japan	G rhiz	A
*Cydonia oblonga* Mill.	Caucasus	P scap	A
*Cyperus papyrus* L.	C.S. Africa	H	A
*Dasylirion serratifolium* (Karw. ex Schult. and Schult. f.) Zucc.	Mexico	H	A
*Dendropanax arboreus* (L.) Decne and Planch. f. *variegata*	Mexico	P scap	A
*Deutzia gracilis* Siebold and Zucc.	Japan	P caesp	A
*Deutzia scabra* Thunb.	Japan	P caesp	A
*Dolichandra unguis-cati* (L.) L.G. Lohmann	C.S. America	P lian	A
*Duranta erecta* L.	C. America/N.South America	P caesp/P scap	A
*Elaeagnus × ebbingei* J. Door.	Korea/Japan	P caesp	A
*Elaeagnus × submacrophylla* Servett.	Korea/Japan	P caesp	A
*Eriobotrya japonica* (Thunb.) Lindl.	China	P caesp/P scap	A
*Erythrina crista-galli* L.	S. America	P scap/P caesp	A
*Erythrina herbacea* L.	C. America	P scap	A
*Erythrostemon gilliesii* (Hook.) Klotzsch	S. America	P caesp	A
*Eucalyptus camaldulensis* Dehnh.	Australia	P scap	A
*Eugenia uniflora* L.	S. America	P caesp/P scap	A
*Euonymus japonicus* Thunb.	Korea/Japan	P caesp	A
*Euonymus japonicus* Thunb. f. *aureo-variegatus*	Korea/Japan	P caesp	A
*Euonymus pulchellus* Jacob-Makoj	Korea/Japan	P caesp	A
*Euphorbia dendroides* L.	S. Europe/N. Africat.	P caesp	N
*Euphorbia ingens* E. May. ex Boiss.	E.S. Africa	P caesp/P scap	A
*Euphorbia murielii* N.E.Br.	Sudan	P caesp	A
*Euphorbia tirucalli* L.	Madagascar	P caesp	A
*Euryops pectinatus* (L.) Cass.	South Africa	P caesp	A
*Fallopia baldeschuanica* (Regel) Holub	E.C. Asia	P lian	A
*Feijoa sellowiana* (O. Berg) O. Berg.	S. America	P caesp	A
*Ficus carica* L.	Caucasus/Balkan	P scap/P caesp	N
*Ficus elastica* Roxb. ex Hornem.	S.E. Asia	P scap	A
*Ficus elastica* Hornem. var. *decora* Guillaumin	S.E. Asia	P scap	A
*Ficus maclellandii* King	S.E: Asia	P scap	A
*Ficus rubiginosa* Desf.	E. Australia	P scap	A
*Firmiana simplex* (L.) W. Wight	S.E. Asia	P scap	A
*Fraxinus angustifolia* subsp. *oxycarpa* (M. Bieb. ex Willd.) Franco and Rocha Afonso	C.S. Europe/Caucasus/N.W. Africa	P scap	N
*Fraxinus excelsior* L.	Europe/Caucasus	P scap	N
*Fraxinus ornus* L.	C.S. Europe/Caucasus	P scap	N
*Furcraea selloana* K. Koch	N. South America	P caesp	A
*Ginkgo biloba* L.	China	P scap	A
*Gleditsia triacanthos* L.	C. America	P scap	A
*Grevillea robusta* A. Cunn. ex R. Br.	E. Australia	P scap	A
*Hedera helix* L.	Europe/Caucasus	P lian	N
*Hedera canariensis* Willd.	Canary Islands	P lian	A
*Hesperocyparis harizonica* (Greene) Bartel	C. America	P scap	A
*Hesperocyparis macrocarpa* (Hartw.) Bartel.	W. America	P scap	A
× *Hesperotropsis leylandii* (A.B. Jachs- and Dallim.) Garl. and Jerry Moore	AH	P scap/Pcaesp	AH
*Hibiscus moscheutos* L.	N.C: America	H scap	A
× *Hibiscus rosa-sinensis* L.	AH	P caesp	AH
*Hibiscus syriacus* L.	China/Taiwan	P caesp/P scap	A
*Ilex aquifolium* L.	Europe/N.W.Africa	P caesp/P scap	N
*Jacaranda mimosifolia* D. Don	C. South America	P scap	A
*Jacobaea maritima* (L.) Pelser and Meijden	S. Europe/N.W.Africa	Ch suffr	N
*Jasminum officinale* L.	Caucasus/E.Asia	P caesp/P lian	A
*Jasminum polyanthum* Franch.	China/Myanmar	P caesp/P lian	A
*Juglans regia* L.	Caucasus	P scap	A
*Juniperus chinensis* L. f. *Pfitzeriana Glauca*	E. Asia	P caesp	AH
*Juniperus horizontalis* Moench	N. America	P caesp	A
*Juniperus oxycedrus* L.	W. Medit/N.W. Africa	P caesp	A
*Justicia adhatoda* L.	S.W. Asia	P caesp	A
*Koelreuteria paniculata* Laxm.	China/Korea	P scap	A
*Lagerstroemia indica* L.	S. E. Asia	P scap/P caesp	A
*Lagunaria patersonia* (Andrews) G. Don	E. Australia	P scap	A
*Lantana camara* L.	C. S. America	P caesp	A
*Lantana montevidensis* (Spreng.) Brig.	S. America	P caesp	A
*Laurus nobilis* L.	S. Europe/N Africa	P caesp/P scap	N
*Lavandula angustifolia* Mill.	S. W. Europe	NP	N
*Ligustrum japonicum* Thunb.	E. Asia	P caesp/P scap	A
*Ligustrum japonicum* Thunb. f. *aureo-variegatum*	E. Asia	P caesp/P scap	A
*Ligustrum lucidum* W.T. Aiton	S. E. Asia	P scap	A
*Ligustrum ovalifolium* Hassk.	Korea/Japan	P caesp	A
*Ligustrum sinense* Lour.	S. E. Asia	P caesp	A
*Liquidambar styraciflua* L.	C. America	P scap	A
*Livistona chinensis* (Jacq.) R. Br. Ex Mart..	E. Asia	P scap	A
*Lonicera caprifolium* L.	C.E.Europe	P lian	N
*Loropetalum chinense* (R. Br) Oliv. f. *rubrum* H.T. Chang	S. E. Asia	P caesp	A
*Magnolia grandiflora* L.	C. America	P scap	A
*Mandevilla laxa* (Ruiz and Pav.) Woodson	S. America	P lian	A
*Melaleuca citrina* (Curtis) Dum. Cours.	E. Australia	P scap/P caesp	A
*Melia azedarach* L.	S.E. Asia/Australia	P scap	A
*Metrosideros excelsa* Sol. ex Gaertn.	New Zealand	P scap	A
*Morus alba* L. f. *pendula*	S.E. Asia/C.S. America	P scap	A
*Murraya paniculata* (L.) Jack	S.E. Asia/Australia	P scap/Pcaesp	A
*Musa × paradisiaca* L.	Malaysia	P scap	A
*Myoporum laetum* G. Forst.	New Zealand	P scap/Pcaesp	A
*Myrtus communis* L.	S.E. Caucasus/N.W, Africa	P caesp	N
*Myrtus communis* L. f. *Tarentina*	S.E. Caucasus/N.W, Africa	P caesp	N
*Nandina domestica* Thunb.	China	P caesp	A
*Nerium oleander* L.	S. Europa/Caucasus/S. Asia/NW Africa	P scap/Pcaesp	N
*Nicotiana glauca* Graham.	C. South America	NP	A
*Oenothera lindheimeri* (Engelm. and A. Gray) W.L. Wagner and Hook	C. America	H scap	A
*Olea europaea* L.	S. Europa/Caucasus/S. Asia/ Africa	P scap	N
*Opuntia ficus-indica* (L.) Mill.	Mexico	P succ	A
*Osmanthus × burkwoodii* (Burkwood and Skipwith) P.S. Green	AH	P caesp	AH
*Ostrya carpinifolia* Scop.	S. Europa/Caucasus	P scap	N
*Paeonia* × *suffruticosa* Andrews	China	G rhiz	A
*Parkinsonia aculeata* L.	C.S. America	P scap	A
*Parthenocissus tricuspidata* (Siebold and Zucc.) Planch.	E. Asia	P lian	A
*Parthenocissus quinquefolia* (L.) Planch.	C.N. America	P lian	A
*Passiflora caerulea* L.	S. America	P lian	A
*Persea americana* Mill.	C. America	P scap	A
*Persea indica* (L.) Spreng.	Madeira/Canary Islands	P scap	A
*Petunia* x*atkinsiana* (Sweet) D. Don ex W.H. Baxter	AH	T scap	AH
*Philadelphus inodorus* L.	C. America	NP	A
*Phlomis fruticosa* L.	Italy/Caucasus/Balkans	NP	N
*Phlomis purpurea* L.	Iberian Peninsula/N.W. Africa	NP	A
*Phlomis viscosa* Poir.	Middle East/Turkey	NP	A
*Phoenix canariensis* H. Wildpret	Canary Islands	P scap	A
*Phoenix dactylifera* L.	Arabian Peninsula/Caucasus	P scap	A
*Photinia serratifolia* (Desf.) Kalkman cv*. Red Robin*	China	P caesp	A
*Phyllostachys aurea* (Andrè) Rivière and C. Rivière	China	P caesp	A
*Phyllostachys nigra* (Lodd. ex Lindl.) Munro	China	P caesp	A
*Phytolacca dioica* L.	S. America	P scap	A
*Picea abies* (L.) H. Karst.	Europe	P scap	N
*Picea pungens* Engelm. Cv. *Kosteriana*	C. America	P scap	A
*Pinus canariensis* C. Sm. ex DC.	Canary Islands	P scap	A
*Pinus halepensis* Mill. subsp*. brutia* (Ten.) Holmboe	Italy/Balkan Peninsula/Caucasus	P scap	N
*Pinus halepensis* Mill.	S. Europa/Caucasus/N.W. Africa	P scap	N
*Pinus nigra* J. F. Arnold	S. Europa/N.W. Africa	P scap	N
*Pinus pinea* L.	S. Europe/Caucasus	P scap	A
*Pinus pinaster* Aiton	S.W. Europe/N.W. Africa	P scap	N
*Pistacia lentiscus* L.	S. Europe/Caucasus/N. Africa	P caesp	N
*Pistacia terebinthus* L.	S. Europe/Caucasus/N. Africa	P caesp	N
*Pittosporum tobira* (Thunb.) W.T. Aiton	E. Asia	P scap/NP	A
*Pittosporum tobira* (Thunb.) W.T.Aiton f. *nanum*	E. Asia	P scap/NP	A
*Platanus × hispanica* Mill. ex Münchh.	AH	P scap	AH
*Platanus orientalis* L.	S. Europe/Balkan Pensinsula/Caucasus	P scap	N
*Platycladus orientalis* (L.) Franco	E. Asia	P caesp	A
*Plumbago auriculata* Lam.	South Africa	Ch frut	A
*Polygala myrtifolia* L.	South Africa	NP	A
*Populus alba* L.	Europe/E. Asia/N. Africa	P scap	N
*Populus nigra* L.	Europe/E. Asia/N. Africa	P scap	N
*Portulacaria afra* Jacq.	South Africa	P succ	A
*Prunus cerasifera* Ehrh.	E. Europe/Balkan/ Caucasus	P caesp	A
*Prunus cerasifera* subsp*. pissardii* (Carrière) Dostál	E. Europe/Balkan/ Caucasus	P caesp	A
*Prunus domestica* L.	Caucasus	P scap	A
*Prunus dulcis* (Mill.) D.A. Webb	Caucasus	P scap	A
*Prunus laurocerasus* L.	Balkans/Caucasus/Lybia	P scap/Pcaesp	A
*Prunus mahaleb* L.	C.S: Europe/Caucasus	P caesp	N
*Prunus webbii* (Spach) Vierh.	S. Europe/Caucasus/N.W. Africa	P caesp	N
*Pterocarya fraxinifolia* (Poir.) Spach	Caucasus	P scap	A
*Punica granatum* L.	Caucasus	P caesp	A
*Pyracantha coccinea* M. Roem.	S. Europe/Caucasus	P caesp	N
*Pyrus calleryana* Decne.	E. Asia	P scap	A
*Pyrus spinosa* Forssk.	S. Europe/Caucasus	P caesp	N
*Quercus cerris* L.	S. Europe/Caucasus	P scap	N
*Quercus ilex* L.	S. Europe/Caucasus/N.W. Africa	P scap/Pcaesp	N
*Quercus ithaburensis* subsp*. macrolepis* (Kotschy) Hedge and Yalt.	S. E.Europe/Caucasus	P scap	N
*Quercus petraea* (Matt.) Liebl.	Europe/Caucasus	P scap	N
*Quercus pubescens* Willd.	Europe/Caucasus	P scap	N
*Quercus robur* L.	Europe/Caucasus	P scap	N
*Quercus suber* L.	S.E. Europe/N.W. Africa	P scap	N
*Quercus trojana* Webb	S.E. Europe/Caucasus	P scap	N
*Rhamnus alaternus* L.	S.E. Europe/Caucasus/C.W. Africa	P scap/P caesp	N
*Rhaphiolepis indica* (L.) Lindl.	E. Asia	P caesp	A
*Rhododendron ferrugineum* L.	C.S. Europe	NP	N
*Robinia pseudoacacia* L.	C. America	P scap	A
*Rosa canina* L.	Europe/E. Asia/N.W. Africa	NP	N
*Rosa × hybrida* Vill. cv. *La Sevillana*	AH	NP	AH
*Ruscus aculeatus* L.	S. Europe/Caucasus/C.W. Africa	Ch frut	N
*Sabal palmetto* (Walter) Lodd. ex Schult. and Schult.f.	C. America	P scap	A
*Salix babylonica* L.	E. Asia	P scap	A
*Salvia fruticosa* Mill.	S.E. Europe/Caucasus/Lybia	Ch frut	N
*Salvia greggii* A.Gray	C. America	H scap	A
*Salvia officinalis* L.	C.S: Europe	Ch suffr	N
*Salvia rosmarinus* Spenn.	S. Europe/Caucasus/N. Africa	NP	N
*Schinus molle* L.	S. America	P scap	A
*Schinus terebinthifolia* Raddi	S. America	P scap	A
*Senecio angulatus* L. f.	South Africa	Ch frut	A
*Senecio grandiflorus* P.J. Bergius	South Africa	T scap	A
*Sideroxylon spinosum* L.	N.W. Africa	P scap	A
*Solanum laciniatum* Aiton	S.W. Australia	NP	A
*Spartium junceum* L.	S. Europe/Caucasus	P caesp	N
*Sterlizia nicolai* Regel and Körn.	South Africa	G rhiz	A
*Sterlizia reginae* Banks	South Africa	G rhiz	A
*Styphnolobium japonicum* (L.) Schott	China	P scap	A
*Syagrus romanzoffiana* (Cham.) Glassman	S. America	P scap	A
*Tamarix africana* Poir. var*. fluminensis* (Maire) Baum	S.W. Europe/C.S.W. Africa	P scap/P caesp	N
*Tamarix arborea* (Ehrenb.) Bunge var. *arborea*	E. Africa	P scap	A
*Tamarix hampeana* Boiss. and Heldr.	Sicily/Balkans/Caucasus	P caesp	N
*Tamarix macrocarpa* Bunge	E.W. Africa	P scap	A
*Tamarix meyeri* Boiss.	Caucasus	P scap	A
*Tamarix parviflora* DC.	S.E. Europe/Caucasus	P scap/P caesp	A
*Tamarix rosea* Bunge	Caucasus	P caesp	A
*Tecoma capensis* Lindl.	S. Africa	P lian	A
*Teucrium fruticans* L.	S.Europe/C:W. Africa	P caesp	N
*Thuja occidentalis* L.	C. NE America	P caesp/P scap	A
*Tilia americana* L.	C. NE. America	P scap	A
*Tilia cordata* Mill.	Europe/C.N Asia	P scap	N
*Tilia platyphyllos* Scop.	Europe/Caucasus	P scap	N
*Tilia × europaea* L.	Europe	P scap	N
*Trachelospermum jasminoides* Lem.	E. Asia	P lian	A
*Trachycarpus fortunei* (Hook) Wendl	E. Asia	P scap	A
*Trema micranthum* (L.) Blume	C.S. America	P scap/P caesp	A
*Ulmus laevis* Pall.	Europe/N.W. Asia	**P scap**	A
*Ulmus minor* Mill.	Europe/W. Asia/C.W. Africa	P caesp	N
*Ulmus minor* Mill. subsp. *canescens* Bartolucci and Galasso	S.E. Europe/Caucasus/C.W. Africa	P caesp	N
*Ulmus parvifolia* Jacq.	C.E. Asia	P caesp	N
*Ulmus pumila* L.	C.E. Asia	P scap/P caesp	A
*Vachellia farnesiana* (L.) Wight and Arn.	C.S. America	P caesp	A
*Vachellia karroo* (Hayne) Banfi and Galasso	South Africa	P caesp	A
*Viburnum lucidum* Mill.	S. Europe/Caucasus/C.W. Africa	P caesp	A
*Viburnum rhytidophyllum* Hemsl.	China	P caesp	A
*Viburnum tinus* L.	S. Europe/Caucasus/C.W. Africa	P caesp	N
*Vitex agnus-castus* L.	S. Europe/S.W: Asia/N. America	P caesp	N
*Washingtonia filifera* (T. Moore and Mast.) H. Wendl. ex de Bary	C. America	P scap	A
*Washingtonia robusta* H. Wendl.	C. America	P scap	A
*Wisteria sinensis* (Sims) DC:	China	P lian	A
*Yucca aloifolia* L.	C. America	P caesp	A
*Yucca gigantea* Lem	C. America	P caesp/P scap	A
*Yucca gloriosa* L.	C. America	P caesp	A
*Yucca rostrata* Engelm. ex Trel.	C. America	P caesp/P scap	A
